# A robotic AI-Chemist system for multi-modal AI-ready database

**DOI:** 10.1093/nsr/nwad332

**Published:** 2023-12-27

**Authors:** Shuo Feng, Aoran Cai, Yang Wang, Baicheng Zhang, Qinyu Qiao, Cheng Chen, Song Wang, Jun Jiang

**Affiliations:** Key Laboratory of Precision and Intelligent Chemistry, School of Chemistry and Materials Science, University of Science and Technology of China, China; Key Laboratory of Precision and Intelligent Chemistry, School of Chemistry and Materials Science, University of Science and Technology of China, China; Key Laboratory of Precision and Intelligent Chemistry, School of Chemistry and Materials Science, University of Science and Technology of China, China; Key Laboratory of Precision and Intelligent Chemistry, School of Chemistry and Materials Science, University of Science and Technology of China, China; Key Laboratory of Precision and Intelligent Chemistry, School of Chemistry and Materials Science, University of Science and Technology of China, China; Key Laboratory of Precision and Intelligent Chemistry, School of Chemistry and Materials Science, University of Science and Technology of China, China; Key Laboratory of Precision and Intelligent Chemistry, School of Chemistry and Materials Science, University of Science and Technology of China, China; Key Laboratory of Precision and Intelligent Chemistry, School of Chemistry and Materials Science, University of Science and Technology of China, China

## Abstract

By fusing literature data mining, high-performance simulations, and high-accuracy experiments, robotic AI-Chemist can achieve automated high-throughput production, classification, cleaning, association and fusion of data, and thus develop a multi-modal AI-ready database.

Artificial intelligence (AI) is being increasingly used in chemical research, not only to make accurate predictions, but also to extract hidden physical laws from the data. Massive high-quality data can give AI powerful capabilities, as demonstrated by the successes of AlphaFold and generative pre-trained transformer (GPT). Existing scientific data are multi-source, multi-type, non-quality-assured, decentralized, and difficult to form a synergy, making it the biggest obstacle to the application of AI in the scientific field. How to obtain high-quality scientific big data with uniform standards and broad coverage, and how to establish multi-modal AI-ready databases for training powerful scientific models, are important issues in combining science and AI.

Scientific data mainly comes from literatures, theoretical calculations and experimental measurements. Therefore, the most effective way to establish a large scientific database is to automate the acquisition and examination of data by combining large-scale data mining, high-performance computational simulations, and high-precision robotic experiments. In recent years, pioneers have developed a variety of automated experimental platforms that are able not only to modify experimental conditions to optimize the experiment independently [1], but also to read the information in the literature to design the experimental scheme [2], and to perform theoretical calculations to assist in the analysis during the experiment [3]. At the same time, mobile robots can be used to perform more general instrumentation tasks and achieve automatic fabrication and characterization in complex and diverse experimental environments [4–6]. Recently, a robotic AI-Chemist platform has been developed, which can automatically read chemical literatures, intelligently design experimental processes, and perform the entire process of simulation-synthesis-characterization-testing experiments [5]. Based on this platform, it is expected to achieve high-throughput data acquisition, interactive calibration of theoretical and experimental data, and validation of literatures, and to establish an AI-ready database covering massive scientific data and integrating chemical knowledge. The process of establishing this database can be divided into five steps: high-throughput production, classification, cleaning, association and fusion (see Fig. 1).

**Figure 1.**
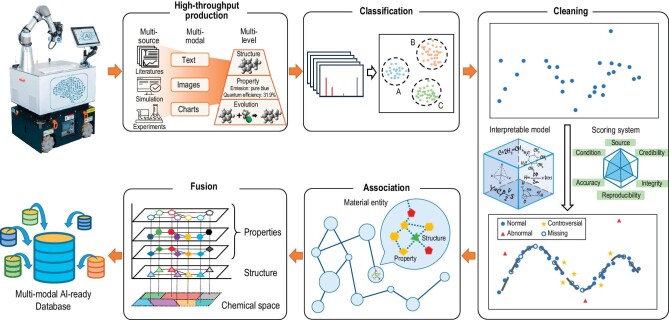
The process for robotic AI-Chemist to establish a large multi-modal AI-ready database.

First, scientific data shall be high-throughput produced from literatures, simulations and experiments. For literatures, the multi-modal data such as text, images, charts are extracted by natural language processing and image recognition technologies. Through interpreting chemical entities and their correlations, the text annotation of spectrograms, tables and chemical symbols can be accomplished. For simulations, it is necessary to automate the construction of material structures and molecular models, and to automatically select appropriate calculation methods to generate large quantities of various physicochemical data. For experiments, unified data formats shall be developed to enable automatic collection and rapid analysis of data, and further to correlate data profiles from different viewpoints of the same sample through different instruments. In this way, robotic AI-Chemist can comprehensively acquire multi-level data on structures, properties, interactions and evolutions, and assign labels and logical semantics to data.

Scientific data obtained from different substances, properties, experimental and computational conditions are not comparable with each other, thus classification is necessary to establish category-based data descriptions. Traditional classification relies on the labelling of data, but scientific data from different fields often lack consistent and comparable labels. Our recent research has shown that spectra are a universal, comparable, theoretically computable, experimentally measurable descriptor, and implicitly contain information about the structure and properties of entities [7]. Spectra-based clustering can well capture the similarity of entities, and classify substances into different categories with significantly different properties [8]. By classifying data from multiple perspectives, such as structural features, spectral features, experimental formulas, fabrication processes and data accuracy, we can precisely define the similarity of data and make comparisons within the same data category.

An AI-ready database also needs to ensure data integrity and accuracy, for which machine-learning models with strong extrapolation capabilities must be established for data cleaning. The first step is to develop intelligent algorithms that can automatically extract reasonable combinations of features as descriptors. For example, spectral descriptors with physical meaning can reflect the similarity and evolution of the structures and properties of substances [9]. Interpretable AI models can construct quantitative mathematical formulas with few parameters by symbolic regression, giving it high robustness, transferability and predictive capability even with imperfect and small data sets [10]. Based on this, we can establish a scoring system that comprehensively evaluates the data in terms of source, credibility, integrity, reproducibility, accuracy, generation conditions, etc. By quantifying the quality of data, eliminating abnormal data points, filling in missing data points, verifying controversial data points through theoretical calculations and robotic experiments, we are able to significantly improve the quality of data.

To achieve the alignment of multi-modal data, data associations need to be established by unifying and standardizing different representations of the same data. Although scientific data are diverse, they share a common material basis, such as different physical properties corresponding to the same molecular conformation and different spectra corresponding to the common vibrational mode. It is possible to take the material entity as the core of association, correlate its attribute data with the entity, and construct an association network between different data by analyzing the relationship between structures, spectra, components and properties. Extracting the common patterns in association network corresponding to the same material basis, we can form alignment criteria for multi-modal data. Furthermore, a knowledge graph can be established by extracting the temporal and logical relationships of entities and events.

Based on the alignment criteria, data fusion can be performed to create a unified, efficient, scalable, structurally unambiguous and multimodal aligned data format, which integrates the characteristics of material structure, properties and reaction features, and is suitable as a unified input for AI models. Combining this database with theoretical simulation and machine-learning models, we can establish digital twin systems for material entities to achieve synergistic evolution of multi-dimensional data in space-time, and accurately predict and optimize the properties and evolutionary processes of matter.

The multi-modal AI-ready database can fuse theoretical and experimental data of matter in different dimensions, provide precise data enriched with material properties and correlations for data-driven research in chemistry, materials science, biology, etc. It can also develop into a universal management system for scientific data, promoting multidisciplinary data exchange and facilitating interdisciplinary collaborations.
